# Electronic Nose-Based Technique for Rapid Detection and Recognition of Moldy Apples

**DOI:** 10.3390/s19071526

**Published:** 2019-03-29

**Authors:** Wenshen Jia, Gang Liang, Hui Tian, Jing Sun, Cihui Wan

**Affiliations:** 1 Beijing Research Center for Agricultural Standards and Testing, Beijing Academy of Agriculture and Forestry Science, Beijing 100097, China; jiaws@brcast.org.cn (W.J.); tianHsandy@126.com (H.T.); mswanc1991@163.com (C.W.); 2 Risk Assessment Lab for Agro-products (Beijing), Ministry of Agriculture, Beijing 100097, China; 3 Beijing Municipal Key Laboratory of Agriculture Environment Monitoring, Beijing 100197, China; 4 Key Laboratory of Urban Agriculture (North China), Ministry of Agriculture and Rural Affairs, Beijing 100097, China; 5 Chinese Academy of Agricultural Engineering, Beijing 100121, China; sunJingHi2008@163.com; 6 Key Laboratory of Agro-Products Postharvest Handling, Ministry of Agriculture, Beijing 100121, China

**Keywords:** electronic nose, apple, mildew, pattern recognition, artificial neural network, nondestructive examination

## Abstract

In this study, the PEN3 electronic nose was used to detect and recognize fresh and moldy apples inoculated with *Penicillium expansum* and *Aspergillus niger*, taking Golden Delicious apples as the model subject. Firstly, the apples were divided into two groups: individual apple inoculated only with/without different molds (Group A) and mixed apples of inoculated apples with fresh apples (Group B). Then, the characteristic gas sensors of the PEN3 electronic nose that were most closely correlated with the flavor information of the moldy apples were optimized and determined to simplify the analysis process and improve the accuracy of the results. Four pattern recognition methods, including linear discriminant analysis (LDA), backpropagation neural network (BPNN), support vector machines (SVM), and radial basis function neural network (RBFNN), were applied to analyze the data obtained from the characteristic sensors, aiming at establishing the prediction model of the flavor information and fresh/moldy apples. The results showed that only the gas sensors of W1S, W2S, W5S, W1W, and W2W in the PEN3 electronic nose exhibited a strong signal response to the flavor information, indicating most were closely correlated with the characteristic flavor of apples and thus the data obtained from these characteristic sensors were used for modeling. The results of the four pattern recognition methods showed that BPNN had the best prediction performance for the training and testing sets for both Groups A and B, with prediction accuracies of 96.3% and 90.0% (Group A), 77.7% and 72.0% (Group B), respectively. Therefore, we demonstrate that the PEN3 electronic nose not only effectively detects and recognizes fresh and moldy apples, but also can distinguish apples inoculated with different molds.

## 1. Introduction

Apples are widely consumed because of their rich vitamin, water, and dietary fiber contents. The apple industry, with an annual total output of more than 40 million tons, has become the first major fruit industry in China. Because of improper storage after harvesting, apples are vulnerable to diseases induced by various internal and external factors, and infections by pathogenic microorganisms, resulting in serious post-harvest losses [1,2]. A few moldy apples, which can be difficult to find from the surface in time, may lead to greater losses in the absence of a prompt solution. Therefore, development of a rapid and nondestructive detection method for moldy apples will help to guarantee the safety and quality of the remaining apples during storage and improve the competitiveness of Chinese apples in the global market. This will help fruit farmers reduce their economic losses and help satisfy consumers by ensuring the quality of the apples.

Methods, such as gas chromatography [3], gas chromatography-mass spectrometry [4], stable isotope identification, and fluorescence spectroscopy, have long been used to analyze the variety and concentrations of single substances in materials under test. However, these methods require expensive instrumentation and fail to comprehensively evaluate the tested materials [5], despite their high accuracy. Most importantly, they are time-consuming and are ineffective for rapid detection applications. The electronic nose technology, which imitates the functions of the human olfactory system, has been rapidly developed. Electronic noses can recognize the characteristic information of complex flavors [6,7] and provide superior performance in terms of response time, detection speed, evaluation range, and repeatability [8]. An electronic nose consists of two parts: a gas sensor array and a pattern recognition system [9,10]. The gas sensor array collects the flavor characteristic information of the sample being tested and sends the resulting data to the pattern recognition system. The pattern recognition system then processes the data and outputs the detection results for specific qualities of the samples. Commonly used pattern recognition methods for electronic noses include principal component analysis (PCA) [11], linear discriminant analysis (LDA) [12,13], support vector machines (SVMs) [14], and artificial neural networks (ANNs) [15,16].

Numerous reports have been published on the application of electronic noses in the detection of different agricultural products. For example, Russo et al. investigated the classification of red onion varieties using the ISE Nose 2000 electronic nose (Airsense Company, Schwerin, Germany) [17]. The analysis accuracy of the deterministic finite automaton (DFA) model used in their research was as high as 97.5%. Konduru et al. tested onions with sour skin disease using an electronic nose composed of nine metal oxide sensors, and the established prediction model attained an accuracy of 85% [18]. Biondi et al. used a PEN3 electronic nose (Airsense Company, Schwerin, Germany) in a survey of common ring rot and brown rot in potatoes [19]. The prediction model in their research achieved a recognition accuracy of 81.3%. Cheng et al. studied tomato seedlings that had been infected with early blight using a PEN2 electronic nose (Airsense Company, Schwerin, Germany) and obtained a model accuracy of 87.5% [20]. Electronic noses have also been applied to mildew detection in grain crops. Yin et al. explored the effects of different features combination characterizations for identifying the moldy maize using a homemade electronic nose [21]. A positive judgment rate of 96.0% was obtained with Fisher discrimination analysis. Lippolis et al. used the ISE Nose 2000 (Airsense Company, Schwerin, Germany) electronic nose to wheat mold detection using the fungal volatile metabolite deoxynivalenol (DON) of wheat as the detection index [22]. The DFA model used in this research yielded a recognition rate of 86.7% for durum wheat.

Researchers have also been working on fruit disease detection using electronic noses [23]. For example, Zhu applied the PEN3 electronic nose to classify and identify strawberries that had been artificially inoculated with three pathogens and obtained satisfactory results [24]. Studies of the application of electronic noses to volatile flavor characteristics information of apples are currently well-established. However, the research mostly focused on differences among varieties [25], freshness identification [26], and storage time prediction of the apples [23,27], along with the detection, analysis, and application of quality and nutrition information [28]. Little attention has been paid to the detection of mildew in apples, especially discrimination of different mildews.

Therefore, inspired by previous research, the PEN3 electronic nose was used to detect and recognize fresh and moldy apples (artificial inoculation with *Penicillium expansum* and *Aspergillus niger* on Golden Delicious apples) in this study. To simplify the analysis process and improve the prediction accuracy, the gas sensor arrays of the PEN3 electronic nose were first optimized and determined. Four pattern recognition methods, including linear discriminant analysis (LDA), backpropagation neural network (BPNN), support vector machine (SVM), and radial basis function neural network (RBFNN), were compared to analyze characteristic flavor data for establishing a prediction model of flavor information for fresh and moldy apples.

## 2. Materials and Methods

### 2.1. Materials

The apples samples used in the experiments were Golden Delicious apples picked from an orchard in the Changping District in Beijing, China. The apples were mature and fresh, all had a similar color, and were without surface damage and diseases. The inoculated molds were *Penicillium expansum* and *Aspergillus niger*; potato agar was used as the culture medium (Sinopharm Chemical Reagent Beijing Co., Ltd., Beijing, China, analytical grade). The PEN3 electronic nose, which has 10 different built-in metallic oxide gas sensors that can detect and identify various common gases (Table 1), was used for collecting the characteristic flavor of apples. All other solutions were prepared with Milli-Q water (18.2 MΩ/cm resistivity) from a Millipore Milli-Q system (Thermo Scientific EASYpure II, Waltham, MA, USA). All glassware ware was pre-washed three times with Milli-Q water and then dried in an oven.

### 2.2. Method of Mold Inoculation

#### 2.2.1. Culture and Purification of the Molds

Firstly, the molds were made into suspensions using the pour plate method; these suspensions were then diluted using sterile water into proportions of 1:10, 1:100, and 1:1000. We used 1 mL from each diluted solution and mixed each solution with sterilized but uncondensed potato agar (acting as the culture medium). After being evenly shaken, the mixed solutions were poured into culture dishes and kept until natural coagulation occurred. The mold plates, created as described above, were then cultured at 25 °C using the plate streak method. Finally, the pure colonies of *Penicillium expansum* and *Aspergillus niger* were obtained about five days later. The filamentous single colonies obtained were then sealed in sterile environments and preserved in a refrigerator at 2 °C for direct use in future experiments.

#### 2.2.2. Mold Inoculation of Apples

Firstly, the apple samples were aired naturally on a sterile workbench after being cleaned using alcohol with purity of 75%. Each apple was then drilled with four holes (3 mm in diameter with a depth of 5 mm) located at different positions on the surface using an inoculation needle. The holes were filled with the pure colonies of *P. expansum* and *A. niger* from the culture dishes using an inoculation ring and were then covered using sterile parafilm (Bemis Company, Inc., Neenah, WI, USA). The inoculated apples were placed into sterile beakers and further sealed using parafilm. The samples were then placed into a thermostat incubator (4 °C) and cultured for five days until mildew appeared. Before the experiments began, the inoculated apples were carefully observed to ensure that they had been impregnated by the molds.

### 2.3. Apple Sample Set Division

Before PEN3 electronic nose measurement, the apple samples were divided into two groups: individual apple only inoculated with/without one kind of mold (single sample group, Group A) and mixed apples of inoculated apples with fresh apples (canned sample group, Group B). The canned sample group consisted of fresh apples and moldy apples (inoculated with single molds of *Penicillium expansum* and *Aspergillus niger*) in a 9:1 ratio, and placed in a sealed can environment. The apple samples of Groups A and B were randomly divided into training sets and testing sets: training set A (TA) and testing set A (VA) for Group A and training set B (TB) and testing set B (VB) for Group B. The training set was used for building the prediction model, and the testing set was used for validating the prediction model. The experimental apple information of different sample groups or sets of fresh or individual molds infected apples is listed in Table 2.

### 2.4. Characteristic Data Collection of Apples Using an Electronic Nose

**Figure 1:**
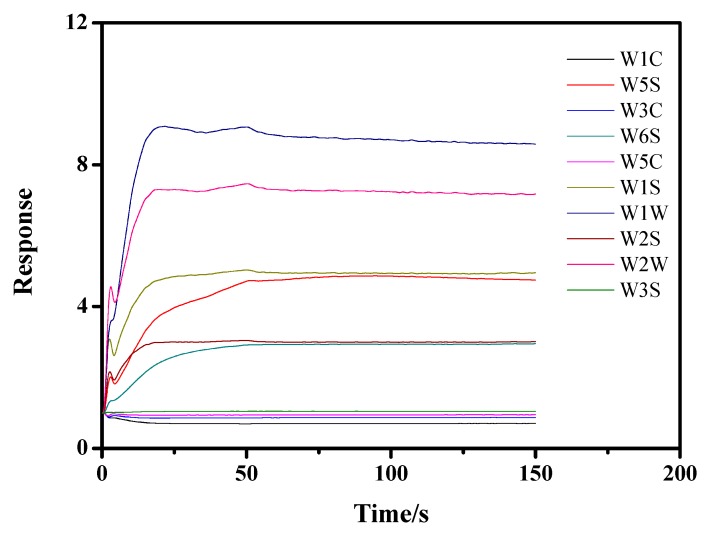
Time-dependent data response of the PEN3 electronic nose.

The PEN3 electronic nose was applied to collect the characteristic flavor of the apples. All the experiments were conducted in the laboratory fume hood. Briefly, the inoculated apples were placed at room temperature (20 °C) for 30 min after they were removed from the incubator. Headspace sampling was then initiated using the PEN3 electronic nose by inserting the sampling and pressure-stabilizing needles into the headspace of the beaker and drawing the flavor from the beaker for 150 s after cleaning for 70 s. Filtered air was used as the carrier gas. Using the PEN3 detection figure, we found that the response value of the sensor tended to be stable after 50 s (Figure 1). Therefore, the data acquired from 60 to 150 s were used as the effective data for the data analysis procedure.

### 2.5. Data Preprocessing

The data preprocessing system is as an important link in the electronic nose system. The sampled odor information signals were transmitted to the data preprocessing system for analysis and processing, producing digital signals (collected sample data). Then, the average value of the data acquired from 60 to 150 s was calculated and used as the feature signal value for statistical analysis. In this study, four pattern recognition methods, including LDA, SVM, BPNN, and RBFNN, were applied to analyze the data obtained from the characteristic sensors. The data analysis for SVM was performed using LibSVM toolbox (Libsvm-3.1) (National Taiwan University, Taipei, Taiwan), and for LDA, BPNN, and RBFNN was performed using MATLAB 2017 (Math works Inc., Natick, MA, USA).

## 3. Results and Discussion

### 3.1. Mold Culture and Inoculation on Apple

**Figure 2:**
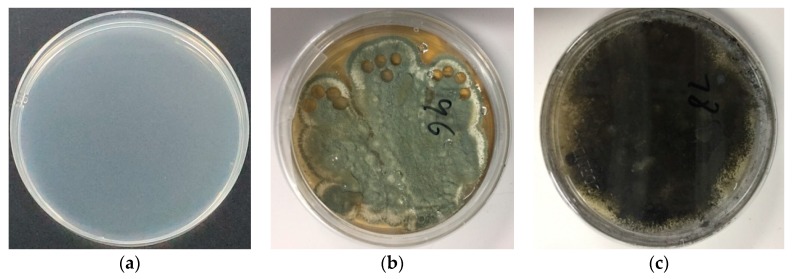
Cultured molds: (**a**) culture medium without mold; (**b**) *Penicilliumex pansum*; and (**c**) *Aspergillus niger*. Conditions: culture at 25 °C for 5 days; dish: 90 mm.

As described in Section 2.2.1., the molds of *P. expansum* and *A. niger* were first cultured in culture dishes with potato agar as the culture medium using the plate streak method. Maintained at 25 °C and five days later, the filamentous single pure colonies of *Penicillium expansum* and *Aspergillus niger* were obtained (Figure 2b and Figure 2c, respectively). The pure colonies obtained were then sealed in sterile environments and preserved in a refrigerator at 2 °C for until use in future experiments.

**Figure 3:**
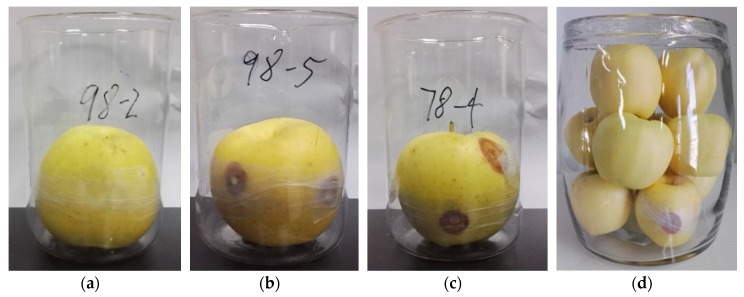
Single apple samples inoculated with different molds: (**a**) no mold (fresh apple); (**b**) *Penicillium expansum*; and (**c**) *Aspergillus niger*; (**d**) Canned apple samples inoculated with single mold (fresh apples: moldy apple = 9:1). Conditions: maintained at 4 °C for 5 days.

Next, the pretreated apples were inoculated with the cultured pure molds by filling the holes drilled in the apple surface using the inoculation ring (the left holes after sampling can be seen in Figure 2). The inoculated apples were placed into sterile beakers, sealed using seal films, and maintained at 4 °C. As shown in Figure 2, the mildew appeared on the apple surface (Figure 3b–d) after five days, which also confirmed the apples were successfully impregnated by the different molds. Then, the inoculated apples were used for PEN3 electronic nose analysis.

### 3.2. Determination of Characteristic Flavorgas Sensors

**Figure 4:**
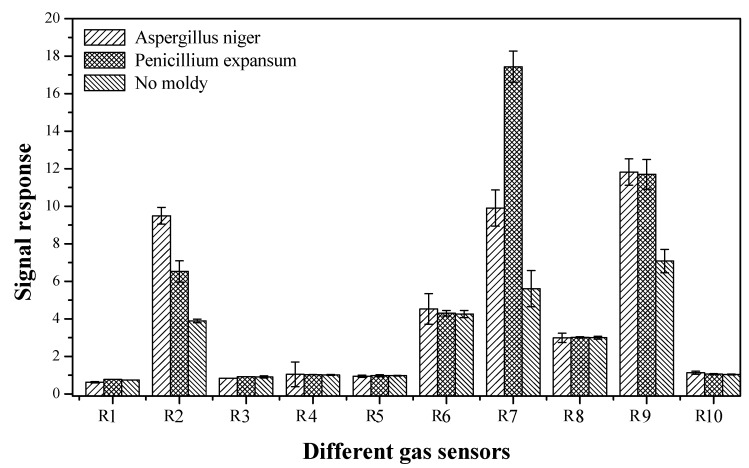
The gas sensors responses of the PEN3 electronic nose to apples inoculated with different molds.

As shown in Table 2, the PEN3 electronic nose has 10 different built-in metallic oxide gas sensors, which simultaneously collect 10 sets of data information in one measurement. However, some of the data information is not characteristic flavor information for fresh and moldy apples. To simplify the subsequent analysis process and improve the prediction accuracy, the gas sensor arrays of the PEN3 electronic nose were first optimized to determine the characteristic flavor sensors for fresh and moldy apples. Then, the response of the PEN3 electronic nose to the fresh and moldy apples (apples inoculated separately with *Aspergillus niger* and *Penicillium expansum*) were explored and the characteristic values were extracted from the data collected by the 10 sensors. As shown in Figure 4, we noticed that the sensors produced different signals for the four sets of samples measured, and only sensors R2, R6, R7, R8, and R9 (W5S, W1S, W1W, W2S, and W2W) exhibited strong responses to the four sets of apple samples, indicating that these sensors are the characteristic flavor gas sensors for apples. Therefore, the data collected from sensors W5S, W1S, W1W, W2S, and W2W were used for data analysis in our study.

### 3.3. Data Analysis

To produce a better recognition rate for the fresh apples and moldy apples (including *Penicillium expansum*-infected apples and *Aspergillus niger*-infected apples), four pattern recognition methods, LDA, BPNN, SVM, and RBFNN, were applied to analyze the data obtained from the optimized characteristic sensors by building the prediction model between flavor information and the fresh and moldy apples. For better comparison, an overview of the recognition rate of the four algorithms for the apples is listed in Table 3.

#### 3.3.1. LDA Analysis

The purpose of LDA is to find a projection that maps the original sample space onto low-dimensional space, so that the projection of high-dimensional data onto low-dimensional space better clusters the samples within the class, and the samples of different categories are separated to the greatest extent. Using the LDA algorithm, the test results of the prediction accuracies were analyzed, as shown in Table 3. Table 3 shows that the LDA algorithm produced prediction accuracies of 79.6% for TA, 68.4% for TA of the training sets, 66.7% for TB, 64.0 for VB of the testing sets. The prediction accuracies were all lower than 80.0%, and even lower than 70% for TA, TB, VA, and VB, indicating that the LDA algorithm showed poor performance. The results also showed that the LDA analysis could not be discriminate the fresh apples, *Penicillium expansum*-infected apples and *Aspergillus niger*-infected apples neither for the single sample group nor for the canned sample group.

#### 3.3.2. SVM Analysis.

SVM is a generalized linear classifier that conducts binary classification on data according to supervised learning, and its decision boundary is the maximum-margin hyperplane that is solved for learning samples. The calculated average values obtained from the characteristic sensors of the PEN3 electronic nose are taken as the input, and the category as the output. As shown in Table 3, the yielded prediction accuracies with the SVM algorithm were 94.4% for TA, 80.0% for VA, 70.5% for TB, and 64.0% for VB. The results indicate that the SVM algorithm exhibited good performance for discrimination of the fresh apples, *Penicillium expansum*-infected apples, and *Aspergillus niger*-infected apples (Group A), but poor recognition rate for the fresh apples, *P. expansum*-infected apples and *Aspergillus niger*-infected apples (Group B). This might be due to the influence of the mixed fresh apples (9:1 ratio) that diluted the flavor information of the mildew-infected apples.

#### 3.3.3. RBFNN Analysis

**Figure 5:**
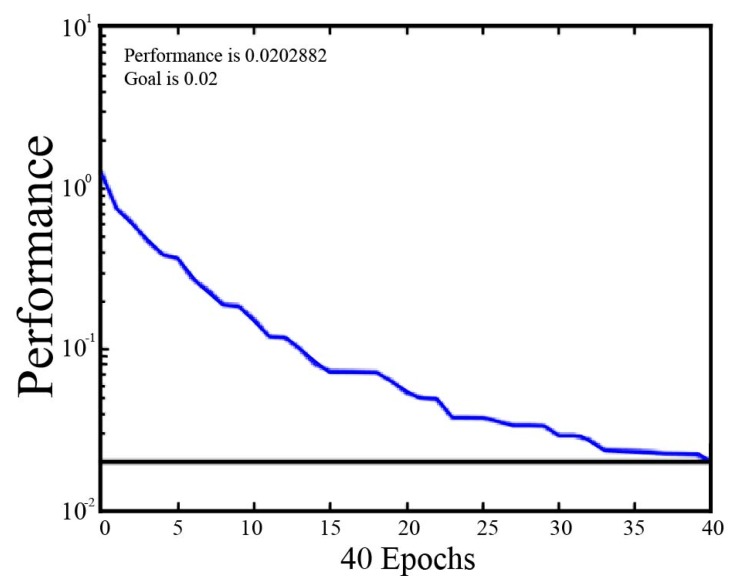
Diagram of the RBFNN iteration process result.

Firstly, the parameters were set for RBFNN analysis. The learning rate was set to 0.08, the momentum factor to 0.1, the maximum iteration epoch to 10,000, and the target accuracy to 0.02. Then, the training data were input into the RBFNN, and the network structure was initialized to train the network. By judging the threshold results of the stable error, adjusting the center and the weight of the hidden layer, and increasing the number of training epochs, the training set was predicted when the error was reduced below the pre-set threshold. The result of the RBFNN iteration process for the moldy apple samples is shown in Figure 5.

As shown in Figure 5, the training error tends to stabilize with a mean square error of 0.020 after 40 training epochs, which meets the maximum error requirements. Constantly increasing the training epochs had little effect on the final recognition results obtained after 40 epochs (data not shown), which was time-consuming. Therefore, the designated training epoch number was set to 40 epochs. Then, the RBFNN models of TA, VA, TB, and VB sets were constructed for both the single sample group (Group A) and the canned sample group (Group B). As shown in Table 3, the RBFNN algorithm yielded prediction accuracies for TA and VA of Group A were all higher than 83.3%, whereas they were all below 71.9% for TB and VB of Group B. The testing accuracy was only 68.0% for VB (a little higher than that of LDA and SVM), indicating that the RBFNN algorithm also exhibited good performance for discrimination of the fresh apples, *Penicillium expansum*-infected apples and *Aspergillus niger*-infected apples (Group A), but showed poor recognition performance for discriminating fresh apples and moldy apples of *Penicillium expansum* and *Aspergillus niger* when mixed with fresh apples.

#### 3.3.4. BPNN Analysis

BPNN exhibits superior performance for data showing non-linear behavior. The learning processes of BPNN are achieved via constant error backpropagation. In this process, the input information propagates forward, while the errors propagate backward. By a process of constant iteration, training is finally suspended when the error accuracy is reduced to below a preset value or the number of training epochs is maximized. The feature values obtained from the characteristic sensors of the PEN3 electronic nose were taken as the input for BPNN analysis, and the results are listed in Table 3. Prediction accuracies higher than 90.0% were obtained for both TA and VA (Group A apple samples) using the BPNN algorithm, indicating the BPNN algorithm exhibited good performance for discrimination of the fresh apples, *Penicillium expansum*-infected apples, and *Aspergillus niger*-infected apples. Prediction accuracies over 70.0% were also achieved for both TB and TB (Group B apple samples), which were all better than the other three algorithms for Group B analysis. From the above discussion, the BPNN algorithm produced the best performance for recognition of fresh and moldy (*Aspergillus niger*, *Penicillium expansum*) apples compared with the other three algorithms, so the BPNN algorithm should be considered as the optimized algorithm for similar applications in future study. 

The results also confirmed that the PEN3 electronic nose provides excellent recognition performance for discriminating fresh and moldy apples, but also a good recognition rate for distinguishing moldy apples inoculated with two different molds (*Aspergillus niger* and *Penicillium expansum*). However, the recognition accuracies of the four algorithms for Group B apple samples (canned sample group) were all lower than those of the Group A apple samples (single sample group), which could explain the collected flavor information of the mildew infected apples being influenced by the mixed fresh apples. The latter was closer to the real storage environment of apples.

## 4. Conclusions

In this study, the PEN3 electronic nose system was used to detect and distinguish fresh and moldy apples inoculated with *Penicillium expansum* and *Aspergillus niger*. The characteristic sensors of the electronic nose system that were most closely correlated with the flavor information of the mildew infected apples were optimized (designated as W5S, W1S, W1W, W2S, and W2W), which simplified the analysis procedure and improved the results accuracy. LDA, BPNN, SVM, and RBFNN were used to model the data obtained from the characteristic sensors, and BPNN provided the best recognition accuracy for the two groups (single sample group and canned sample group), and a higher recognition accuracy was produced for the single sample group. Therefore, the findings in this study prove that the PEN3 electronic nose can effectively detect and recognize fresh and moldy apples, and can distinguish moldy apples inoculated with *Penicillium expansum* and *Aspergillus niger*. Further study is still needed to obtain a high recognition rate for fresh apples, *Penicillium expansum*-infected apples, and *Aspergillus niger* for the canned sample group. Electronic nose technology can meet the demands for rapid, low-cost, and nondestructive detection, which provides references for developing apple mildew detection equipment in the future.

## Figures and Tables

**Table 1:** Performance of the sensor arrays of the PEN3 electronic nose.

No. in Array	Sensor Name	Reaction Compound	Typical Target
R1	W1C	Aromatic compounds	C_6_H_5_CH_3_
R2	W5S	Oxynitride	NO_2_
R3	W3C	Aromatic constituents, mainly ammonia	C_6_H_6_
R4	W6S	Hydrogen	H_2_
R5	W5C	Alkanes, aromatic compounds	C_3_H_8_
R6	W1S	Broad Methane	CH_4_
R7	W1W	Sulfides and organic sulfides	H_2_S
R8	W2S	Broad alcohols	C_2_H_5_OH
R9	W2W	Aromatics, organic sulfides	H_2_S
R10	W3S	Alkanes, especially methane	CH_4_

**Table 2:** Experimental apple information of different sample groups.

Sample Group		Training Set		Testing Set	
		Training Samples	Number of Apples	Training Samples	Number of Apples
Group A	Fresh	54	54	10	10
	*Penicillium expansum*	54	54	10	10
	*Aspergillus niger*	54	54	10	10
Group B	Fresh	49	490	9	90
	*Penicillium expansum*	45	450	8	72 ^a^ 8 ^b^
	*Aspergillus niger*	45	450	8	72 ^a^ 8 ^b^

^a^ fresh apples; ^b^ moldy apples.

**Table 3:** Recognition accuracies of the four algorithms for the two groups of apples.

Sample Group	Algorithm	Recognition Rate of Training Set	Recognition Rate of Testing Set
Group A	LDA	79.6%	66.7%
	SVM	94.4%	80.0%
	RBFNN	88.9%	83.3%
	BPNN	96.3%	90.0%
Group B	LDA	68.4%	64.0%
	SVM	70.5%	64.0%
	RBFNN	71.9%	68.0%
	BPNN	77.7%	72.0%

LDA-linear discriminant analysis; SVMs-support vector machines; RBFNN-radial basis function neural network; BPNN-backpropagation neural network.
